# A validated, transitional and translational porcine model of hepatocellular carcinoma

**DOI:** 10.18632/oncotarget.18872

**Published:** 2017-06-29

**Authors:** Kyle M. Schachtschneider, Regina M. Schwind, Kwame A. Darfour-Oduro, Arun K. De, Lauretta A. Rund, Kuldeep Singh, Daniel R. Principe, Grace Guzman, Charles E. Ray, Howard Ozer, Ron C. Gaba, Lawrence B. Schook

**Affiliations:** ^1^ Department of Animal Sciences, University of Illinois, Urbana, IL, USA; ^2^ Animal Breeding and Genomics Centre, Wageningen University, Wageningen, The Netherlands; ^3^ Department of Radiology, University of Illinois at Chicago, Chicago, IL, USA; ^4^ Veterinary Diagnostic Laboratory, University of Illinois, Urbana, IL, USA; ^5^ College of Medicine, University of Illinois at Chicago, Chicago, IL, USA; ^6^ Department of Pathology, University of Illinois at Chicago, Chicago, IL, USA; ^7^ Department of Medicine, Division of Hematology/Oncology, University of Illinois at Chicago, Chicago, IL, USA

**Keywords:** hepatocellular carcinoma, human, porcine model, gene expression, interventional radiology

## Abstract

Difficult questions are confronting clinicians attempting to improve hepatocellular carcinoma (HCC) outcomes. A large animal model with genetic, anatomical, and physiological similarities to humans is required to transition from mouse models to human clinical trials to address unmet clinical needs. To validate our previously reported inducible porcine cancer model (Oncopig) as a transitional HCC model, Oncopig hepatocyte cultures were transformed using Cre recombinase. The resulting porcine HCC cells (pHCC) expressed oncogenic *TP53*^R167H^ and *KRAS*^G12D^, and displayed nuclear pleomorphisms with pale to granular cytoplasm arranged in expanded plates similar to human HCC histopathology. Human HCC transcriptional hallmarks were detected in pHCC cells using RNA-seq, including *TERT* reactivation, apoptosis evasion, angiogenesis activation, and Wnt signaling activation. Master regulators of gene expression were conserved across Oncopig and 18 human HCC cell lines. pHCC injection into SCID mice resulted in tumors recapitulating human HCC characteristics, including thick trabeculae formation, pseudoacini patterning, and sheets of well-vascularized stroma. Finally, autologous injection of pHCC cells subcutaneously yielded a tumor histologically characterized as Edmondson Steiner (HCC nuclear grade assessment system) grade 2 HCC with trabecular patterning and T-lymphocyte infiltration. These data demonstrate the Oncopig HCC model's utility for improving detection, treatment, and biomarker discovery relevant to human HCC.

## INTRODUCTION

The overall 5-year survival rate of hepatocellular carcinoma (HCC) is 17.5% with more than half of cases diagnosed with regionally advanced disease, distant metastases, or unknown stages that carry relative survival rates of 10.9%, 3.1%, and 6.1% according to the National Cancer Institute Surveillance, Epidemiology, and End Results Program (NCI SEER) stat fact sheets, respectively (https://seer.cancer.gov/statfacts/html/livibd.html; accessed 2017). Unfortunately, due to tumor characteristics, underlying liver disease and patient comorbidities, the vast majority of HCC patients are not candidates for surgery with curative intent, leaving at least 85% of patients to consider alternative palliative therapies [1]. Sorafenib is currently the only FDA approved targeted systemic therapy for advanced HCC and provides, on average, only 12 weeks of additional survival benefit with no difference in the median time to symptomatic progression [2]. Thus, there is an urgent need for novel and more effective treatment strategies. Treatment of HCC is complex, and there is limited high-level evidence for comparative evaluation of the many different non-surgical management approaches. This is further complicated by new technologies and locoregional therapies (LRTs) that are continually evolving. There are several medical specialists involved in the diagnosis and treatment of HCC, and treatment strategies are often dictated by the specialty of the consulting physician, rather than evidence-based consensus [3]. The critical need for an optimal HCC treatment strategy is demonstrated by previous clinical trials and the more than 1,000 related ongoing trials (https://www.clinicaltrials.gov/; accessed 2017).

The Oncopig cancer model (OCM) is a transgenic pig with Cre recombinase inducible porcine transgenes encoding *KRAS*^G12D^ and *TP53*^R167H^, a commonly mutated signal transducing oncogene and tumor suppressor found in over 50% of human cancers, respectively [4]. Expression of these transgenes following exposure to adenovirus encoding Cre (AdCre) results in cell transformation leading to development of site, cell, and temporally specific tumors [4]. The OCM is of ideal size since it allows for utilization of the same tools that are used in human clinical practice. The size of the pig and its similarity to humans in anatomy, physiology, immunogenetics, and epigenetics [5] make it an ideal platform to develop an animal model that recapitulates human HCC and associated co-morbidities, e.g., obesity, cirrhosis, and nonalcoholic steatohepatitis (NASH). Pigs also require multiple genetic changes to develop cancer, and human mutations introduced into pig genes recapitulate molecular pathways associated with human cancers [4, 6]. Furthermore, there is increasing evidence that intra-arterial therapies can stimulate cytokine production that may, in fact, drive tumor progression [7, 8]; thus, evaluating the role of concurrent targeted and systemic therapies is essential. The OCM can support studies aiming to satisfy various endpoints, including: overall survival, progression free survival, and cost-effectiveness.

Changes in cytological features, the production of specific proteins, and alterations in gene expression and pathway regulation characterize human HCC. Transcriptional hallmarks of HCC include limitless replicative potential resulting from reactivation of *TERT*, evasion of apoptosis, and activation of *VEGFA*, *PDGFA*, or *ANGPT2*, resulting in neoangiogenesis. Disturbances in cell cycle regulation due to *RB1* silencing or *TP53* mutations, as well as activation of the Wnt signaling pathway are also commonly observed in human HCC [9, 10]. The goal of this study was to validate the OCM as a model for human HCC in terms of phenotype, gene expression, and tumor development. Importantly, these factors are not yet considered when planning HCC therapy, however, we envision the OCM as a pivotal tool for defining this space. We hypothesized that the OCM mimics human HCC at the histologic and molecular level, providing an ideal transitional and translational research platform for improving detection, treatment, biomarker discovery, and other unmet clinical needs for HCC.

## RESULTS

### Oncopig hepatocyte cultures recapitulate characteristics of human hepatocyte cultures

**Figure 1:**
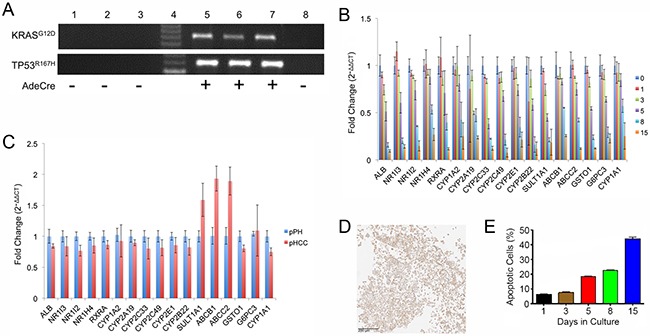
Hepatocyte gene expression and survival in culture **(A)** Agarose gel electrophoresis of RT-PCR products for transgenes (*TP53*^R167H^ and *KRAS*^G12D^). Lane 1, pPH1 cell line; Lane 2, pPH2 cell line; Lane 3, pPH3 cell line; Lane 4, Molecular weight markers; Lane 5, pHCC1 cell line; Lane 6, pHCC2 cell line; Lane 7, pHCC3 cell line; Lane 8, Oncopig fibroblasts (negative control). **(B)** Expression of drug metabolism enzyme, nuclear receptor, and hepatocyte functional genes in pPH cells over time as assessed by qPCR. Numbers represent days in culture. **(C)** Relative expression of pHCC cells (passage 8) cultured in the presence of DMSO compared to pPH cells after 1 day of culturing as assessed by qPCR. **(D)** Positive Hep Par-1 staining of cultured pHCC cells (scale bar 200 μm). **(E)** Proportion of pPH cells positive for Annexin-V staining (apoptotic) detected by Flow Cytometry. Bar graphs indicate the average across the cell lines, with error bars representing the standard deviation.

Oncopig primary hepatocyte (pPH) cell lines from three Oncopigs were cultured and transformed (pHCC) by exposure to AdCre *in vitro*, resulting in expression of mutant *KRAS*^G12D^ and *TP53*^R167H^ transgenes in pHCC but not pPH cells (Figure 1A). The pPH cell lines displayed expression of important drug metabolic enzymes, regulatory genes, and hepatocyte functional genes that reduced over time in culture (Figure 1B), consistent with reports in human primary hepatocytes [11]. Also consistent with previous human HCC cell line studies [11], pHCC cells cultured with DMSO expressed similar levels of the same drug metabolism and hepatocyte functional genes as pPH cells (Figure 1C). In addition, Hep Par-1 positive staining (hepatocyte marker; >90%) was observed in all three pHCC cell lines (Figure 1D). Finally, while the number of apoptotic pPH cells rose from 6.45% on day 1 to 44.23% on day 15 of culture (Figure 1E), pHCC cells were still viable after 130 passages. Together, these results confirm the identity of the pPH and pHCC cells as primary and transformed hepatocytes, respectively.

### Oncopig pHCC cells recapitulate human HCC characteristics

**Figure 2:**
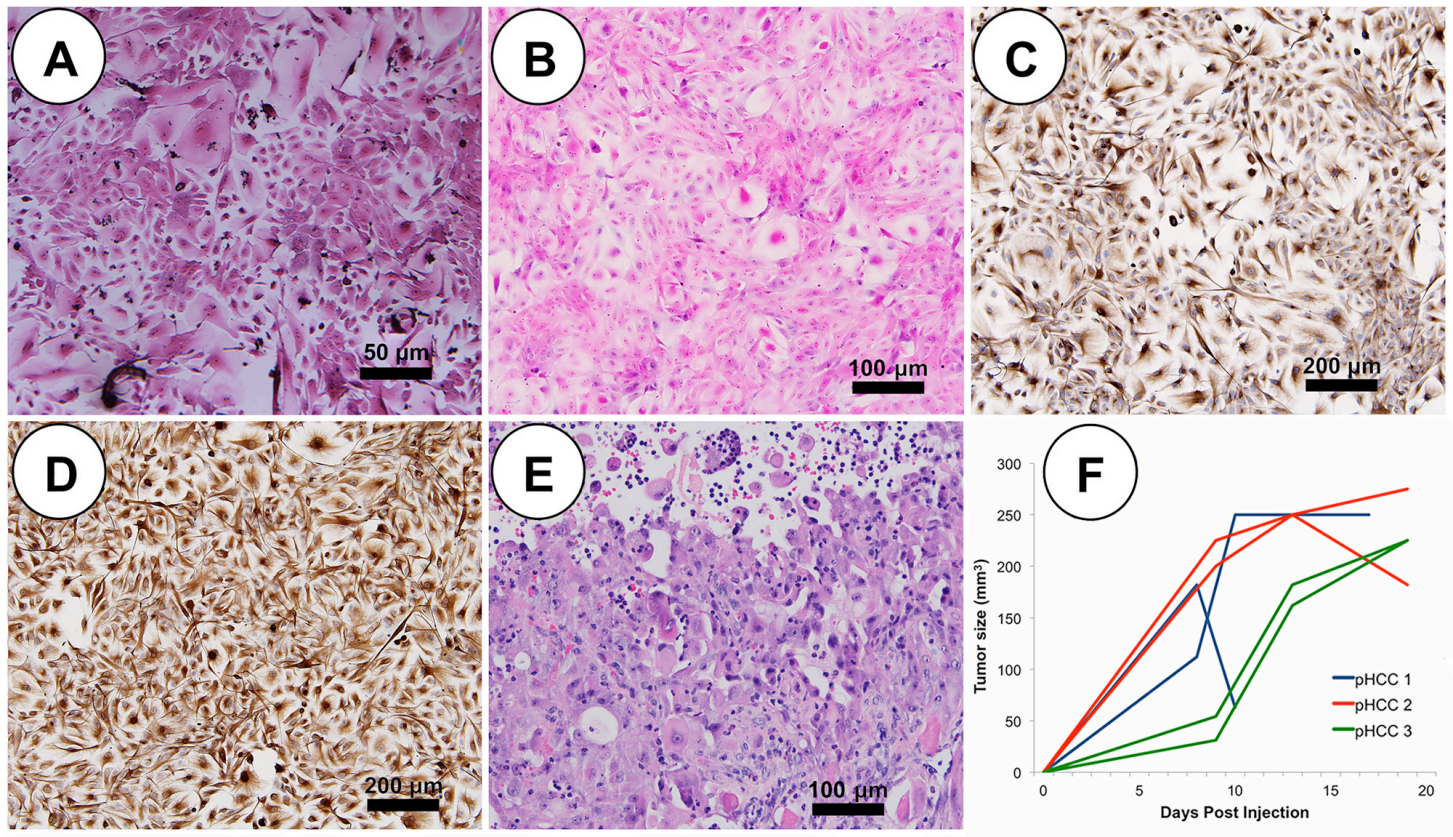
pHCC recapitulates cytologic and histologic features of human HCC both *in vitro* and *in vivo* **(A)** Representative pPH cells in culture show polygonal shape with granular cytoplasm (scale bar 50 μm). **(B)** In culture, pleomorphic elongated pHCC have clear to granular cytoplasm and round to oval pleomorphic nuclei (scale bar 50 μm). **(C)** Positive cytokeratin immunostaining of cultured pHCC cells (scale bar 50 μm). **(D)** Positive vimentin immunostaining of cultured pHCC cells (scale bar 50 μm). **(E)** H&E stained SC xenografted tumor (scale bar 50 μm). **(F)** Growth curve of SC xenografted tumors indicative of linear growth kinetics.

Cytopathological features of human HCC cells include round to polygonal and eosinophilic or granular cytoplasm with round to oval nuclei and prominent nucleoli [12]. pPH cells displayed bright and translucent cytoplasm with well-contrasted borders (Figure 2A). In contrast, blind assessment by a board-certified human pathologist with subspecialty training in Liver and Transplantation Pathology revealed pHCC cells displayed cytopathological characteristics similar to human HCC cells, including nuclear hyperchromatism pleomorphism, increased nuclear cytoplasmic ratio, and round to oval pale eosinophilic or granular cytoplasm (Figure 2B). Immunohistochemistry was utilized to determine whether pHCC cells underwent an epithelial-mesenchymal transition (EMT), a common tumor progression signal observed in human HCC. While both pPH and pHCC cells expressed cytokeratin (epithelial marker; Figure 2C), on average 80% of pHCC cells expressed vimentin (mesenchymal marker; Figure 2D), confirming pHCC cells have undergone an EMT. The pHCC cells also secreted alpha-fetoprotein (AFP; 40 to 50 ng/ml), a serum marker observed in 60-70% of human HCC, whereas pPH cell supernatants contained no measurable amount of AFP after 48 hours (detection limit 3.12 ng/ml), further demonstrating recapitulation of core cytological features of human HCC.

### Oncopig HCC tumor phenotype

**Figure 3:**
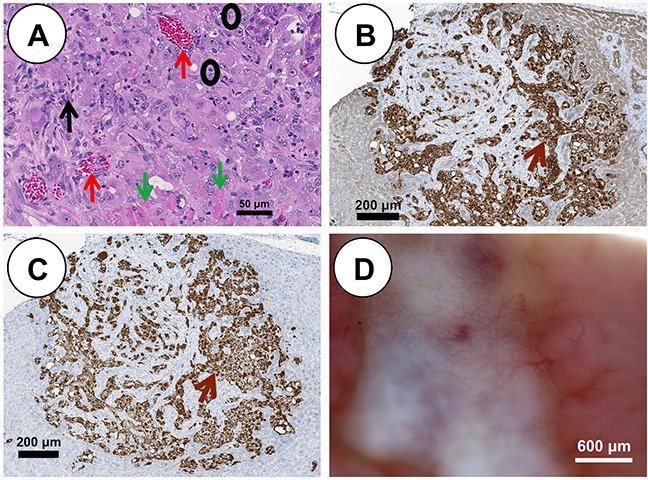
pHCC intrahepatic xenografted tumors recapitulate cytologic and histologic features of human HCC **(A)** H&E stained pHCC intrahepatic xenografted tumor reveals human HCC characteristics including blood vessels (red arrows), stroma (black arrow), neoplastic cells (black circles) and necrotic cells (green arrows) at 21 days post injection (scale bar 50 μm). **(B)** Positive cytokeratin immunostaining (brown arrow) of pHCC intrahepatic xenografted tumor (scale bar 200 μm). **(C)** Positive vimentin immunostaining (brown arrow) of pHCC intrahepatic xenografted tumor (scale bar 200 μm). **(D)** Blood vessel development in pHCC intrahepatic xenografted tumor (scale bar 600 μm).

Each Oncopig pHCC cell line was injected subcutaneously (SC) and into the hepatic parenchyma of severe combined immunodeficiency (SCID) mice developing tumors within 21 days. SC tumors were histomorphologically characterized by expansive and infiltrative neoplastic cells with classical histologic features of human HCC [9], including arrangement of neoplastic cells in more than 3 cell layer thick trabeculae with regional pseudoacinar and sheet formations supported by a thick wall of vascularized stroma (Figure 2E). Consistent with previous *in vivo* tumor growth characterization studies [13], pHCC SC xenografted tumors exhibited a linear growth curve (R^2^ = 0.688, p < 0.00001; Figure 2F and Supplementary Figure 1). pHCC intrahepatic xenografted tumors were also histomorphologically characterized by classical histological features of human HCC, including arrangement of neoplastic cells in more than 3 cell layer thick expanded liver cell plates with regional pseudoacinar and sheet formations supported by a thick wall of vascularized stroma (Figure 3A), as well as expression of cytokeratin (Figure 3B) and vimentin (Figure 3C) consistent with the EMT observed in human HCC. Moreover, intrahepatic xenografted tumors displayed significant angiogenesis similar to human HCC (Figure 3D).

**Figure 4:**
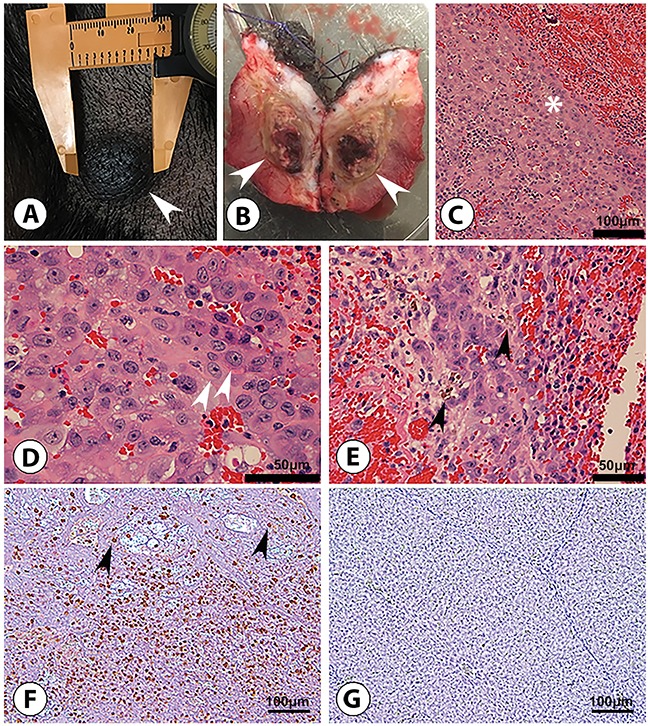
Subcutaneous HCC formation in an Oncopig following autologous transfer of pHCC cells **(A)** 3 cm right flank nodule (arrowhead) after 14 days of tumor growth, at which point the nodule was excised. **(B)** Excised 2.7 cm nodule (46 days post injection) displays transected ovoid subcutaneous tumor (arrowheads). **(C-E)** H&E examination shows malignant cells consistent with Edmondson-Steiner grade 2 HCC; these include a trabecular (ribbon-like) pattern of malignant cells (* in panel C; scale bar 100 μm), characterized by acidophilic and granular cytoplasm, ovoid to round hyperchromatic nuclei, and increased nuclear to cytoplasmic ratio (arrowheads in panel D; scale bar 50 μm) containing wispy brown material (arrowheads in panel E; scale bar 50 μm) representing bile typical of hepatocyte differentiation. **(F)** Anti-pig CD3 staining reveals diffuse CD3 T-lymphocyte (arrowheads) infiltration into tumor, indicating a “hot” tumor potentially susceptible to immunotherapy (scale bar 100 μm). **(G)** Anti-pig CD3 staining of control (non-tumorous) Oncopig liver tissue displays no CD3 T-lymphocyte presence (scale bar 100 μm).

Autologous transplantation (SC) of pHCC cells induced a palpable mass in a single Oncopig, detected at 4 days post injection at the middle and high dose injection sites, and 19 days post injection at the low dose site. The diameter of the high dose mass reached 3 cm by day 14 (Figure 4A). The 2.7 cm mass was recovered at euthanasia (46 days post injection; Figure 4B) and blindly described histologically as Edmondson Steiner grade 2 human HCC with trabecular patterning (Figure 4C–4E). Masses at the middle and low dose sites were no longer palpable by 27 days post injection. Furthermore, diffuse T-lymphocyte infiltration into the autologous tumor was detected by immunohistochemistry (Figure 4F and 4G).

### Oncopig HCC gene expression profiles

**Figure 5:**
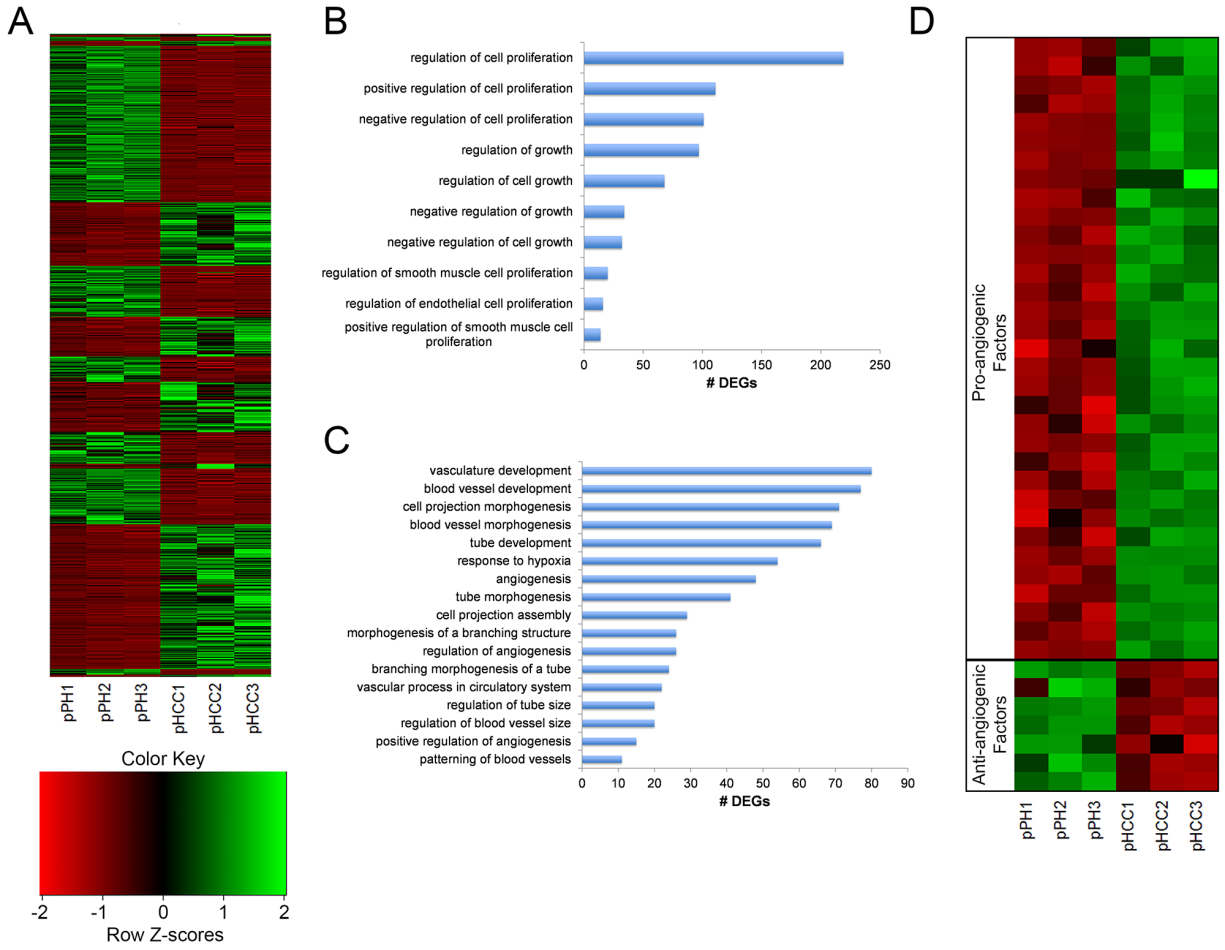
pHCC cell line expression related to cell cycle progression and angiogenesis **(A)** Heat map of normalized expression levels of 3,481 differentially expressed genes (DEGs) for individual cell lines, represented as z-scores. **(B)** Enriched gene ontology (GO) terms associated with regulation of cell growth and proliferation. **(C)** Enriched GO terms associated with regulation of angiogenesis. **(D)** Heatmap of pro- and anti-angiogenic factors displaying elevated and reduced expression in pHCC cell lines, respectively, represented as z-scores.

Transcriptional hallmarks of human HCC include: 1) disturbances in cell cycle regulation as a result of *RB1* silencing or *TP53* mutations; 2) sustained angiogenesis resulting from overexpression of *VEGFA*, *PDGFA*, or *ANGPT2*; 3) evasion of apoptosis; and 4) reactivation of *TERT* [10]. These transcriptional features were assessed in pPH and pHCC cell line gene expression profiles via RNA-seq. A total of 3,481 differentially expressed genes (DEGs) were identified between the pHCC and pPH cells, with 1,792 and 1,689 genes displaying elevated and reduced expression in the pHCC cells, respectively (Supplementary Tables 1 and 2). As expected, increased *TP53* and *KRAS* expression was observed in pHCC cells (Supplementary Table 1), with the majority of transcripts originating from the mutant transgenes (ratio of mutant:WT reads of 17.55:1 and 3.40:1 for *TP53* and *KRAS*, respectively). In addition, expression profiles were more highly correlated within groups (Pearson's r = 0.97-0.98, p < 0.0001) than across groups (Pearson's r = 0.84-0.89, p < 0.0001; Supplementary Figure 2). Samples clustered by group when comparing the relative expression of all DEGs (Figure 5A), demonstrating the reproducible effect of mutant transgene expression on gene expression profiles.

**Table 1:** Master regulators of genes with reduced expression in Oncopig and human HCC cell lines

Transcription factors	Oncopig pHCC	7703	Focus	Hep3B	Hep3B-TR	Hep40	HepG2	HLE	HLF	HUH-1	HUH-6	HUH-7	SK-Hep1	SNU-182	SNU-387	SNU-389	SNU-449	SNU-475	PLC/PRF/5
*STAT1*	769	-	-	-	-	-	-	-	-	-	-	-	-	-	-	-	-	-	-
*EP300*	607	431	456	450	438	596	307	545	392	343	379	385	488	270	462	664	652	626	410
*FOXA2*	541	384	418	394	396	553	298	477	362	312	349	218	451	245	431	638	577	561	386
*SPI1*	535	-	-	-	-	-	-	-	-	-	-	-	-	-	-	-	-	-	-
*FOXA1*	532	448	478	441	452	630	324	559	408	368	398	382	506	286	506	712	657	667	449
*HNF4A*	522	435	463	421	429	556	304	542	383	347	387	370	477	285	436	672	654	644	408
*HNF4G*	391	408	424	421	378	550	293	500	364	331	351	382	458	248	445	628	597	584	390
*CEBPB*	337	372	384	380	276	357	33	442	339	221	235	252	419	244	408	553	527	535	366
*HNF1A*	274	-	-	-	-	-	167	-	330	-	-	-	-	-	-	-	-	-	-
*NFIC*	234	215	226	122	179	169	-	257	208	173	114	172	243	128	228	186	179	291	205
*HDAC2*	223	353	396	369	206	498	120	451	320	157	147	161	396	236	387	497	442	489	346
*NR2F2*	174	152	149	137	141	183	95	179	141	123	129	124	170	80	153	218	204	198	148
*NR3C1*	156	-	-	91	-	-	-	-	-	-	-	157	-	-	-	-	-	-	-
*FOXA3*	115	-	-	-	-	-	-	-	-	-	-	-	-	-	-	-	-	-	-
*GATA3*	102	-	-	-	-	-	69	-	-	-	-	-	-	-	-	-	-	-	-
*E2F1*	77	-	-	-	-	-	-	-	-	-	-	-	-	-	-	-	-	-	-
*STAT2*	41	-	-	-	-	-	41	-	-	-	-	-	-	-	-	-	-	-	-

The number of target genes with reduced expression for each transcription factor is indicated for each cell line. (−) Indicates that transcription factor target genes were not enriched (i.e. overrepresented) in the list of genes with reduced expression for a given cell line.

### Activation of TP53 dependent cell cycle progression

Uncontrolled proliferation through altered cell cycle regulation as a result of *RB1* silencing or *TP53* mutation represents a key transcriptional feature of human HCC [10]. Gene ontology (GO) terms involved in regulation of cell growth and proliferation were enriched for DEGs in the pHCC cells (Figure 5B). The combined enrichment of these terms and mutant *TP53^R167H^* expression led to the hypothesis that *TP53* dependent upregulation of cyclin D and B, factors promoting G1-to-S and G2-to-M transitions, led to increased cell cycle progression. As expected, reduced expression of cyclin D inhibitors *SFN* and *GADD45G* was observed (Supplementary Table 2), resulting in increased expression of cyclin D genes *CCND1* and *CCND2* (Supplementary Table 1). In addition, increased expression of *CCNB3* was also observed (Supplementary Table 1). *RB1* silencing was not observed in the pHCC cells, suggesting activation of mutant *TP53^R167H^* is the main driver of cell cycle progression in pHCC cells.

### Expression of pro-angiogenic factors

Increased angiogenesis is essential for tumors to receive nutrients, and hypervasularity resulting from overexpression of *VEGFA*, *PDGFA*, or *ANGPT2* is a key hallmark of human HCC [14, 15]. *ANGPT2* primes the vasculature for angiogenic response in the presence of *VEGFA* [16], which induces angiogenesis [17] and is upregulated by *PDGFA* [18]. Consistent with the observed hypervascularity of intrahepatic pHCC tumors (Figure 3D), GO terms involved in regulation of angiogenesis were enriched for DEGs in pHCC cells (Figure 5C). In addition, elevated expression of *PDGFA* and *ANGPT2* was observed (Supplementary Table 1), as well as elevated and reduced expression of a number of pro- and anti-angiogenic factors, respectively (Figure 5D).

### Evasion of apoptosis

**Figure 6:**
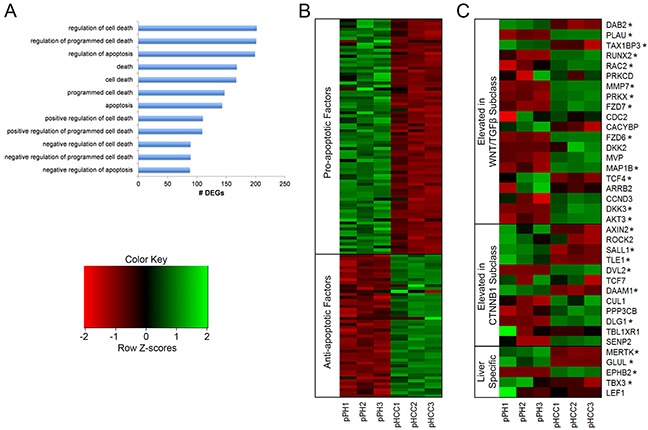
Gene expression related to apoptosis and Wnt signaling in pHCC cell lines **(A)** Gene ontology (GO) terms enriched for differentially expressed genes (DEGs) associated with regulation of apoptosis. **(B)** Heatmap of pro- and anti-apoptotic factors displaying reduced and elevated expression in pHCC cell lines, respectively, represented as z-scores. **(C)** Expression profiles of genes whose elevated expression is used to classify human HCC, indicating pHCC cell lines represent the WNT/TGFβ subclass, represented as z-scores. * denotes q < 0.05.

Altered regulation of genes involved in apoptosis results in evasion of apoptosis in cancer cells, and was observed in the pHCC cells through enrichment of GO terms involved in the regulation of apoptosis (Figure 6A). A number of anti-apoptotic factors in these pathways were highly expressed (Figure 6B), including *TWIST1* (Supplementary Table 1), which protects cells from oncogene-induced apoptosis [19] and is overexpressed in human HCC [20]. In addition, many pro-apoptotic factors were lowly expressed (Figure 6B), including those expressed at low levels in human HCC, such as *TNFSF10*, which induces apoptosis in cancer but not normal cells [21], and *STAT1*, which mediates the growth inhibition and apoptotic activities of IFN-γ [22]. *ATF5* induces apoptosis in response to the chemotherapy drug cisplatin [23] and was also lowly expressed in pHCC cells (Supplementary Table 2).

### Activation of telomere maintenance

The *TERT* gene encodes for telomerase, an RNA polymerase that adds telomere repeat sequences at the end of chromosomes. As in humans, the porcine *TERT* gene is silenced in somatic cells, resulting in telomere shortening over time [9], eventually resulting in cell death. *TERT* is expressed in cancer cells to achieve limitless replicative potential [24], and increased *TERT* expression is observed in 90% of human HCC [25]. Consistent with human HCC, elevated *TERT* expression was observed in pHCC cells (Supplementary Table 1), indicating activation of telomere maintenance.

### Subclass specific Wnt signaling activation

Activation of Wnt signaling promotes increased HCC cell proliferation, migration, and invasion [26] through binding of Wnt ligands to frizzled receptors, resulting in translocation of β-catenin into the nucleus. Wnt ligand and frizzled receptor overexpression indicates aberrant Wnt signaling activation and is detected in over half of HCC cases [27–30]. Increased expression of Wnt ligands *WNT2B*, *WNT9A*, and *WNT10B* was observed in pHCC cells (Supplementary Table 1). Elevated expression of the frizzled receptors *FZD6* and *FZD7*, as well as the co-receptor *LRP6* was also observed (Supplementary Table 1). In addition, HCC tumors can be classified based on Wnt related gene expression [31]. The Wnt/TGFβ subclass is characterized by an aggressive phenotype and elevated expression of classical Wnt target genes, while the *CTNNB1* subclass is characterized by a less aggressive phenotype and elevated expression of liver specific Wnt target genes. Elevated expression of 10 genes related to the Wnt/TGFβ subclass was observed in the pHCC cells, 8 of which are involved in Wnt pathway activation, compared to 2 for the *CTNNB1* subclass (Figure 6C). The *CTNNB1* subclass is also characterized by increased expression of 9 liver-related Wnt target genes, 5 of which are annotated in the pig genome. Elevated expression of only one of these (*EPHB2*) was observed in pHCC cells, while 3 displayed reduced expression (Figure 6C). Together, these results provide evidence of Wnt signaling activation in pHCC cells resembling the Wnt/TGFβ subclass of human HCC.

### Master regulators of Oncopig and human HCC cell line expression

Master regulators are transcription factors (TFs) that play a key role in regulating gene expression. To determine whether the same TFs underlie genome-wide expression changes in human and Oncopig HCC, transcriptional regulatory networks were reverse-engineered from Oncopig and human HCC cell line gene expression profiles, resulting in the identification of TFs underlying altered expression of co-expressed genes. Master regulators of reduced gene expression were highly conserved, with 8 TFs (*EP300*, *HNF4G*, *HDAC2*, *NR2F2*, *FOXA1*, *HNF4A*, *CEBPB*, and *FOXA2*) identified in the Oncopig and all human HCC cell lines (Table 1). In addition, *EP300* target genes were the most highly enriched in both the Oncopig and the majority of human (13/18) HCC cell lines. Although master regulators driving increased gene expression were identified in the Oncopig pHCC cells (Supplementary Table 3), the same TFs were not identified in the human HCC cell lines. Unlike the master regulators of reduced gene expression, none of the identified master regulators driving increased gene expression were shared across all 18 human HCC cell lines (Supplementary Table 4). No master regulator of increased gene expression was shared across more than 16 human HCC cell lines, highlighting the high level of variability in regulation of elevated compared to reduced gene expression in human HCC.

### Independent validation of RNA-seq results

To validate the RNA-seq expression results, genes displaying elevated and reduced expression were randomly chosen for expression analysis using qPCR. The directional change in expression for each gene was consistent between RNA-seq and qPCR (Supplementary Table 5). In addition, a strong correlation (Pearson's r = 0.994, p value < 0.0001) was observed between the log2 fold change differences obtained using RNA-seq and qPCR, demonstrating the reliability of the reported RNA-seq results.

### Cirrhosis induction

**Figure 7:**
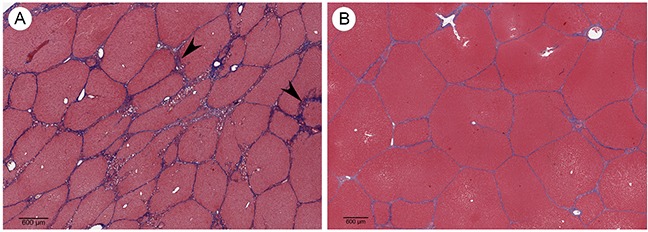
Cirrhosis induction **(A)** Trichrome stained Oncopig liver 8 weeks following cirrhosis induction shows irregular and morphologically effaced hepatic lobules circumferentially surrounded by thick fibrous septa (arrowheads) consistent with stage 4 liver fibrosis (cirrhosis; scale bar 600 μm). **(B)** Trichrome stain of normal Oncopig control liver shows morphologically non-expanded portal tracts maintaining their usual hexagonal distribution, without cetrilobular venular and sinusoidal thickening (scale bar 600 μm).

As HCC often develops in patients presenting with HCC risk factors such as cirrhosis, an ideal HCC model must also be able to reflect comorbidities associated with HCC development. In order to demonstrate the ability of the OCM to model relevant comorbidities, cirrhosis induction was performed on 2 Oncopigs via transarterial alcohol injection. This procedure resulted in histologic changes—including irregular and morphologically distorted hepatic lobules circumferentially surrounded by thick fibrous septa—consistent with stage 4 liver fibrosis (cirrhosis) within 8 weeks (Figure 7).

## DISCUSSION

There are a number of difficult questions confronting clinicians and clinical investigators attempting to improve HCC outcomes. The existence of numerous competing phase II and phase III clinical trials impairs accrual to all trials because of limited patient availability. Prioritization of trials with substantial scientific rationale for advancing new therapeutic agents or procedures for HCC through the clinical pipeline, from phase I through III has become critical. Clearly a relevant, biologically analogous animal HCC model would accelerate therapeutic discovery while offering a more efficient, cost effective mechanism for therapeutic evaluation of this deadly disease.

The OCM was designed and developed to provide a large animal transitional, translational, and transformative model for accelerating clinical trials and supporting noninvasive image-guided procedures, radiation oncology, drug metabolism, immune therapy of cancer, surgical training, and technology development (e.g., early detection and screening) [32–37]. The large size of the pig and its similarity in anatomy, physiology, metabolism, genetics, and epigenetics to humans make it an ideal research and development platform. The OCM carries a Cre recombinase inducible porcine *KRAS^G12D^* and *TP53^R167H^* construct, a commonly mutated oncogene and tumor suppressor gene in human cancers, respectively. This study validates the *hypothesis* that genetic alteration of molecular pathways in the pig, commonly disrupted in human cancers, provides a standardized histologic tumor model analogous to human tumors to support new investigations of therapeutic interventions.

The ability to induce cirrhosis in the OCM provides the opportunity to assess the role of cirrhosis in tumorigenesis through development of pHCC lines from normal and cirrhotic livers. In addition, because patients with vascular invasion and cirrhosis carry an 83% likelihood of recurrence, versus 12% of patients presenting without vascular invasion and cirrhosis [38], the OCM provides a unique opportunity to stage HCC with and without cirrhosis to define mono- or combination therapeutic approaches in a more controlled way than can be done in human patients. However, although cirrhosis was successfully modeled in the OCM, intra-hepatic tumors were not developed herein. This limitation requires that further studies be undertaken to produce orthotopic tumors via engraftment of SC HCC tumors into the liver before this level of modeling can be achieved. Finally, while autochthonous HCC tumors have been developed in a previously described chemically induced porcine HCC model [39, 40], this model takes over 1 year to develop clinically relevant tumors and does not allow for control of tumor number, location, or comorbidities, rendering it potentially less suitable for preclinical and co-clinical trials.

The results presented here demonstrate the Oncopig HCC model recapitulates core cytological phenotypic characteristics of human HCC, resembles poorly differentiated human HCC with cellular pleomorphy, and affords an opportunity to utilize an animal model that recapitulates human HCC presenting with poor prognosis. Critical to the utilization of animal models in studying human diseases is the reproducibility of experiments. In this study, independently derived cell lines obtained from Oncopigs from 2 different litters demonstrated reproducible human HCC characteristics and gene expression profiles, providing evidence for the OCM as a robust, validated, and consistent model of human HCC. The pHCC cell lines also secrete AFP, regarded as the most useful serum protein biomarkers for HCC detection in humans [41]. In high- risk populations, intermittent AFP serum levels and liver ultrasounds are utilized as surveillance strategies, but as HCC is detected at a localized stage in fewer than 50% of cases, there remains a need for more reliable screenings (https://www.cancer.gov/types/liver/patient/liver-screening-pdq; accessed 2017). The secretion of AFP by pHCC cells suggests that the Oncopig HCC model mimics AFP-producing HCC, with the advantage that AFP levels can be used to monitor tumor progression and treatment response in the Oncopig in drug trials for later application to human HCC. In addition, since the OCM depends on the same cumulative genetic mutations and mimics key transcriptional hallmarks of human HCC, there exists the potential for biomarker discovery using serum and liver samples at various reproducible stages of tumorigenesis.

Tumor development resulting from engraftment of Oncopig pHCC cells into mouse livers recapitulated phenotypic features of human HCC. This was also the case for tumors produced via autologous injection of Oncopig pHCC cells SC. Although xenografted tumors typically exhibit a Gompertz or exponential-linear growth curve [13], a linear growth curve was observed for the SC xenografted tumors described in this study. While surprising, it is likely that the exponential growth phase was missed in this study due to the relatively few number of tumor measurements taken (2-4/tumor over a 3 week period) compared to the number recommended for assessment of high growth rate tumors (3x/week) [42]. It is unclear at this time whether the palpable masses at the middle and low dose autologous transfer sites represent tumors that regressed, or masses caused by an inflammatory response and subsequent clearing of the pHCC cells before a tumor mass could be established. In either case, the successful formation of an HCC tumor at the high dose site indicates that autologous tumor formation is feasible, and that additional studies are required to confirm reproducibility.

Of clinical relevance was the significant angiogenesis exhibited by the Oncopig HCC tumors as opposed to rodent models. Human HCC tumors are typically hypervascular [43], making them amenable to anti-angiogenic gene therapy. These results, in addition to the altered expression of genes involved in angiogenesis regulation, suggest the Oncopig HCC model is more suitable for testing LRTs and anti-angiogenic therapies than commonly used rodent models. However, it is important to note that while hypervascularization of Oncopig HCC tumors and elevated expression of proangiogeneic factors in pHCC cell lines was observed, orthotopically engrafted tumors may not mimic the vascularity of autochthonous tumors, which may influence the relevance of this model for locoregional therapy studies. Therefore, further studies are required to determine the hypervascularity of orthotopically engrafted Oncopig HCC tumors.

Immunotherapy trials utilizing immune checkpoint inhibitors have become a major focus of research for cancer treatment. In order for these treatments to be effective, tumors must demonstrate high levels of lymphocyte infiltration prior to treatment. These tumors presenting with significant lymphocyte infiltration are referred to as “hot” tumors. Tumor infiltrating T-lymphocytes were identified in the Oncopig HCC tumors presented here, indicating these are “hot” tumors potentially utilizable for immunotherapy trials. Although characterization of T-cell populations and tumor cell immunogenicity has been performed in other Oncopig tumor types (Overgaard N.H. et al, 2017 AACR Annual Meeting Abstract #2587), future studies are required to further characterize the immune environment of Oncopig HCC tumors.

It was further demonstrated that pHCC cells closely parallel human HCC at the transcriptomic level in terms of *TP53* dependent cell cycle regulation and *CCND1* overexpression, increased angiogenesis through elevated *PDGFA* and *ANGPT2* expression, evasion of apoptosis, reactivation of *TERT* allowing uncontrolled cellular replication, and Wnt signaling activation. Furthermore, Oncopig pHCC expression profiles most closely resemble the Wnt/TGFβ subclass of human HCC, a subclass characterized by an aggressive HCC clinical phenotype. Given the focus on identification of key pathways for the development of targeted human HCC treatments, the OCM provides a suitable model in which novel targeted therapies, such as those targeting the Wnt pathway, can be tested before transitioning to clinical trials.

A number of the master regulators of reduced gene expression identified across Oncopig and human HCC cell lines have been previously implicated in HCC development. *HDAC2* is a transcriptional repressor whose expression promotes cell cycle progression by down regulating genes involved in G1 cell cycle arrest in human HCC [44], while *HNF4A* promotes cellular proliferation and loss of epithelial morphology when lowly expressed in human HCC [45]. In addition, *FOXA2* overexpression inhibits migration and invasion in human HCC cell lines [46], while *EP300* expression is associated with an aggressive HCC phenotype [47], highlighting the importance of these TFs in HCC patient prognosis. Given the previous implication of these master regulators in HCC development, the role of the other master regulators identified across Oncopig and human HCC cell lines (*NR2F2*, *HNF4G*, *FOXA1* and *CEBPB*) warrant further investigation. In conclusion, the identification of consistent pathway alterations and master regulators of genes displaying reduced expression across Oncopig and human HCC cell lines further validates the OCM as a model for human HCC.

Although master regulators of reduced gene expression were conserved across Oncopig and the 18 human HCC cell lines, master regulators of elevated gene expression were not. In addition to not being conserved across species, none of the master regulators driving increased gene expression were shared across all 18 human HCC cell lines. This result highlights the high level of variability in regulation of elevated compared to reduced gene expression in human HCC, and helps explain the lack of shared master regulators of elevated gene expression between Oncopig and human HCC cell lines. It is important to note that this study utilized expression profiles from pHCC cell lines, and although these profiles recapitulated many of the transcriptional hallmarks of human HCC, further *in vivo* expression profiling is required to further confirm the similarities observed between Oncopig and human HCC cell lines.

In summary, we present data demonstrating the ability of Oncopig pHCC cell lines to recapitulate key features of human HCC, providing validation for the use of the OCM for human HCC research. This model will enable the testing of different HCC treatment techniques and the evaluation of histologic, radiographic, and pathological responses in a model more similar to human HCC than currently available small animal models.

## MATERIALS AND METHODS

### Animals

All animal procedures were approved by The University of Illinois Institutional Animal Care and Use Committee (IACUC protocol number, 14125, 14126, and 15121). All Oncopigs were crossbred animals (Minnesota Minipig sire and Yorkshire dams) and heterozygous for the transgene. In total, 6 Oncopigs and 15 SCID mice were used in this study.

### pPH isolation and transgene activation

pPH cells were isolated from 3 individual Oncopigs utilizing a modified Meng's method [48] to produce 3 individual pPH lines. A portion of a liver lobe was collected from each Oncopig, washed 2-3 times with ice cold phosphate buffer saline (PBS) and placed in ice cold Krebs Ringer Solution. Liver samples were cannulated into visible blood vessels on the cut surface and flushed with 500 ml of buffer A (8.3 g/l NaCl, 0.5 g/l KCl, 2.4 g/l HEPES, and 0.19 g/l EGTA at pH 7.4) at 37°C, followed by perfusion of 500 ml buffer B (8.3 g/l NaCl, 0.5 g/l KCl, and 2.4 g/l HEPES) at 37°C. Tissues were further perfused using a pre-warmed (37°C) digestion Buffer C (3.9 g/l NaCl, 0.5 g/l KCl, 2.4 g/l HEPES, 0.7 g/l CaCl2 × 2H2O, and 0.1 % Collagenase, type IV). Following digestion, the liver capsules were removed and dissolved cells were liberated by gently shaking the liver specimen in ice cold buffer D (9.91 g/l HBSS without calcium and magnesium, 2.4 g/l HEPES, and 2.0 g/l bovine serum albumin). Regions that were not well perfused were cut with a scalpel to release additional hepatocytes. The resulting cell suspensions were subjected to serial filtering to decrease contamination of other cell types through size-specific exclusion. Cell suspensions were first filtered through a 100 μm nylon mesh (BD Falcon, Franklin Lakes, NJ, USA) and centrifuged at 50 x g for 3 min at 4°C. Thereafter, cells were incubated for 10 min with DNase1 containing buffer at 4°C to break down cell clumps and digest damaged cells. The resulting suspensions were filtered through a 70 μm nylon mesh (BD Falcon, Franklin Lakes, NJ, USA) and centrifuged at 50 x g for 3 min. Cell pellets were washed three times with ice cold buffer D before re-suspension. 2×10^6^ cells were seeded in 5 ml culture medium (William's E supplemented with 100 mU/ml penicillin, 100 μg/ml streptomycin, 2mM glutamate and 10% Fetal bovine serum) in T25 culture flasks. 4 to 6 hours following seeding culture medium and unattached cells were discarded and replaced with fresh medium. Medium was replaced every 24 hours for the duration of pPH culturing, and pPH cell lines were never passed.

### pPH cell transformation

On day 2 of culture, the medium for each of the pPH cell lines was changed to low serum (5% FBS) and Ad5CMVCre-eGFP (AdCre; University of Iowa Vector Core, Iowa City, IA, USA) was added at a 200 to 500 MOI as previously described [4]. Following AdCre treatment, as the resulting pHCC cells reached confluency, the medium was removed and cells were detached by incubating with trypsin until the cells became detached following agitation of the flask. Cells were passed by plating at 1:2 dilution into new T25 culture flasks in 5 ml culture medium. At passage 4 the pHCC medium was transitioned to DMEM supplemented with 100 mU/ml penicillin, 100 ug/ml streptomycin, 2mM glutamate and 10% Fetal bovine serum.

### DMSO treatment of the pHCC cells

The pHCC cells were seeded at low density (2 × 10^4^ cells/cm^2^) in supplemented William's E (10% FBS, 100 mU.mL^−1^ penicillin, 100 μg.mL^−1^ streptomycin, 2 mM glutamate). At confluence, the medium was supplemented with 2% DMSO. The medium was changed every 2-3 days. The cells were cultured in presence of DMSO for 8 passages before collecting for RT-PCR analysis.

### Apoptosis assay

Flow cytometric quantification of pPH apoptosis was performed on day 1, 3, 5, 8, and 15 of culture. The FITC Annexin V/Dead Cell Apoptosis Kit (Invitrogen, Life Technologies, Carlsbad, CA, USA) was used following the manufacturer's protocol. pPH cells were harvested, washed in PBS, and stained with Annexin V-FITC and propidium iodide. The cells were incubated at room temperature for 15 min and fluorescence was measured by flow cytometry (BD LSR II Flow Cytometer, BD Biosciences, San Jose, CA, USA). The data was analyzed using FCS Express 4 software. One-way analysis of variance (ANOVA) followed by Dunnett post-test was performed to determine significant difference among treatments.

### AFP secretion

Cell lines were cultured for 48 hours in 5 ml of medium and AFP levels for 1×10^6^ plated cells were determined using a porcine AFP ELISA kit (MyBioSource, Inc, San Diego, CA, USA).

### Xenograft tumorigenesis assays

pHCC cell lines (passage 9) were evaluated for tumorigenicity by injecting 2.5×10^6^ cells (in 100 μl of Matrigel, BD Biosciences, San Diego, CA, USA) SC into 6 SCID (NOD.CB17-Prkdcscid/ JAX, Bar Harbor, ME, USA) female mice (2 sites/cell line). Tumor growth data was subjected to multiple linear regression analysis using StatPlus software. Following verification of pHCC tumorigenicity by day 21 post injection (monitoring by palpation), cells (1×10^6^ cells in 25 μl Matrigel) were injected into the liver parenchyma of 9 SCID female mice (3 sites/cell line). After 21 days, animals were euthanized and evaluated for tumor growth.

### Autologous tumorigenesis assay

An additional pHCC cell line was established using a surgically resected liver from a single Oncopig (112 days of age) and transformed as described above. At 81 days post resection pHCC cells (passage 9) were harvested, washed 3 times with serum free media, diluted to a total volume of 1 ml DMEM, and autologously injected into 3 SC sites (1.0 x10^7^, 1.25×10^6^ or 7.8×10^4^ per site). Injection sites were monitored visually, by palpation, and by biopsy. On day 46 post injection the animal was euthanized and tissues collected for further analysis.

### Immunocytochemistry

Cytological and histomorphological features of cell lines and tumors were evaluated by H&E, Hep Par-1 (Leica Biosystems, Wetzlar, Germany), vimentin (Biocare Medical, Concord, CA, USA), cytokeratin (Biocare Medical, Concord, CA, USA), and CD3 (Santa Cruz Biotech, Santa Cruz, CA, USA) staining as previously described [4]. Staining was performed at passage 9 for pHCC cells and culture day 2 for pPH cells.

### Transgene expression

*KRAS*^G12D^ and *TP53*^R167H^ transgene expression was determined using RT-PCR as previously described [4]. RNA from previously isolated Oncopig primary fibroblasts [4] were used as a negative control.

### Transcriptomic analysis

RNA-seq was performed on Oncopig cell lines as previously described [4], adjusting the -mate-inner-dist option to 50 and the - mate-std-dev option to 450 in the alignment step, and utilizing cuffdiff for differential analysis. An average of 29 million raw stranded paired-end reads were produced for each cell line (Supplementary Table 7). The datasets are available in the European Nucleotide Archive under accession number PRJEB8646, samples ERS1210942-ERS1210947 (www.ebi.ac.uk/ena/data/view/PRJEB8646). Genes orthologous to human genes with log2 fold change ≥1 or ≤-1 and a q value ≤0.05 were considered differentially expressed. Gene transcription levels are represented as fragments per kilobase of transcript per million mapped reads (FPKM). GO term and pathway enrichment analysis for DEGs was performed as previously described [5], using all genes as a background.

### qPCR

Expression of important drug metabolism enzymes, regulatory genes, and hepatocyte functional genes were determined in pPH (day 0, 1, 3, 5, 8, and 15 of culture) and pHCC cells cultured in DMSO (passage 8) using qPCR. In addition, RNA-seq results were validated by qPCR using randomly chosen genes displaying elevated and reduced expression in the RNA-seq datasets. qPCR was performed using the Power SYBR green PCR Master Mix (Applied Biosystems, Foster City, CA, USA) as previously described [4]. The thermal cycling conditions were one cycle of 50°C for 2 min and 95°C for 10 min, followed by 40 cycles of 95°C for 15 sec and 60°C for 1 min. Fold changes were determined using the 2^−ΔΔCt^ method by normalizing to *GAPDH* and *ACTB*. Primers are provided in Supplementary Table 6.

### Identification of master regulators

Master regulators of gene expression were identified in Oncopig and 18 human HCC cell lines [49] using the cytoscape plugin iRegulon v1.3 [50]. iRegulon utilizes expression data to uncover regulatory relationships through reverse-engineering of gene regulatory networks, allowing for the identification of TFs underlying altered expression of co-expressed genes. Specifically, iRegulon utilizes a list of co-expressed genes and cis-regulatory sequence information from multiple species to identify TFs underlying genome-wide expression changes. Relative expression levels for human HCC cell lines were extracted from http://medicalgenomics.org/cellminerhcc, and genes with a log2 fold change >1 or <-1 relative to controls were considered differentially expressed. DEGs displaying elevated and reduced expression were analyzed separately for each cell line to identify master regulators of genome-wide increases and decreases in expression, respectively. TF binding motifs with a normalized enrichment score >3 were considered significantly enriched, which corresponds to a false discovery rate (q-value) between 3% and 9% [50].

### Cirrhosis induction

Liver cirrhosis was induced in 2 Oncopigs as previously described [51]. Procedures were undertaken following induction of general anesthesia. Briefly, with the Oncopig in a supine position, the groin area was sterilely prepped and draped. Femoral arterial access was gained via ultrasound guided vascular access, with placement of a 5 French vascular sheath (Pinnacle; Terumo, Somerset, NJ, USA). Using standard catheter and wire techniques, celiac arteriography was performed using a 5 French catheter (GLIDECATH; Terumo, Somerset, NJ, USA). A coaxial 3 French microcatheter (Renegade Hi-flo; Boston Scientific, Natick, MA, USA) was then advanced into the proper hepatic artery, and an emulsion of absolute ethanol and ethiodized oil (Lipiodol; Guerbet, Villepinte, France) (1:3 v/v dosed at 0.75 mL/kg) was slowly infused by manual injection into the hepatic arterial circulation over approximately 45-60 minutes. All devices were then removed, and hemostasis was obtained by direct compression at the vascular access site. Assessments were performed by a human pathologist according to the METAVIR system [52].

## SUPPLEMENTARY MATERIALS FIGURE AND TABLES



## Abbreviations

AdCre: adenovirus encoding Cre
AFP: alpha-fetoprotein
DEGs: differentially expressed genes
FPKM: fragments per kilobase of transcript per million mapped reads
GO: gene ontology
HCC: hepatocellular carcinoma
LRTs: locoregional therapies
NASH: nonalcoholic steatohepatitis
OCM: Oncopig cancer model
PBS: phosphate buffer saline
pHCC: porcine HCC
pPH: porcine primary hepatocytes
SC: subcutaneous
SCID: severe combined immunodeficiency
TFs: transcription factors
