# Supplementary material for: A validated, transitional and translational porcine model of hepatocellular carcinoma
Source: Oncotarget. 2017 Jun 29;8(38):63620–34. doi: 10.18632/oncotarget.18872 (PMC5609948; doi:10.18632/oncotarget.18872)
Supplement: Supplementary file 1 [file oncotarget-08-63620-s001.pdf]

A validated, transitional and translational porcine model of hepatocellular carcinoma

SUPPLEMENTARY MATERIALS

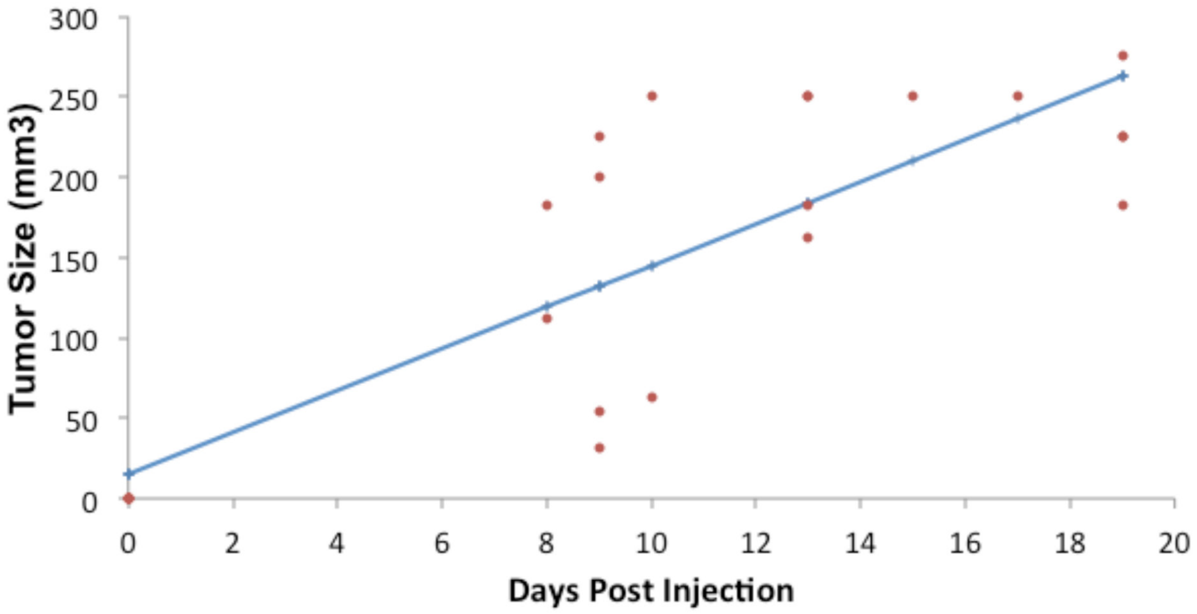

Supplementary Figure 1: Linear fit plot of SC xenografted tumor growth. Scatter plot of SC xenografted tumor growth indicative of relatively linear growth kinetics. A line fit plot is a scatter plot for the actual data points along with the fitted regression line.

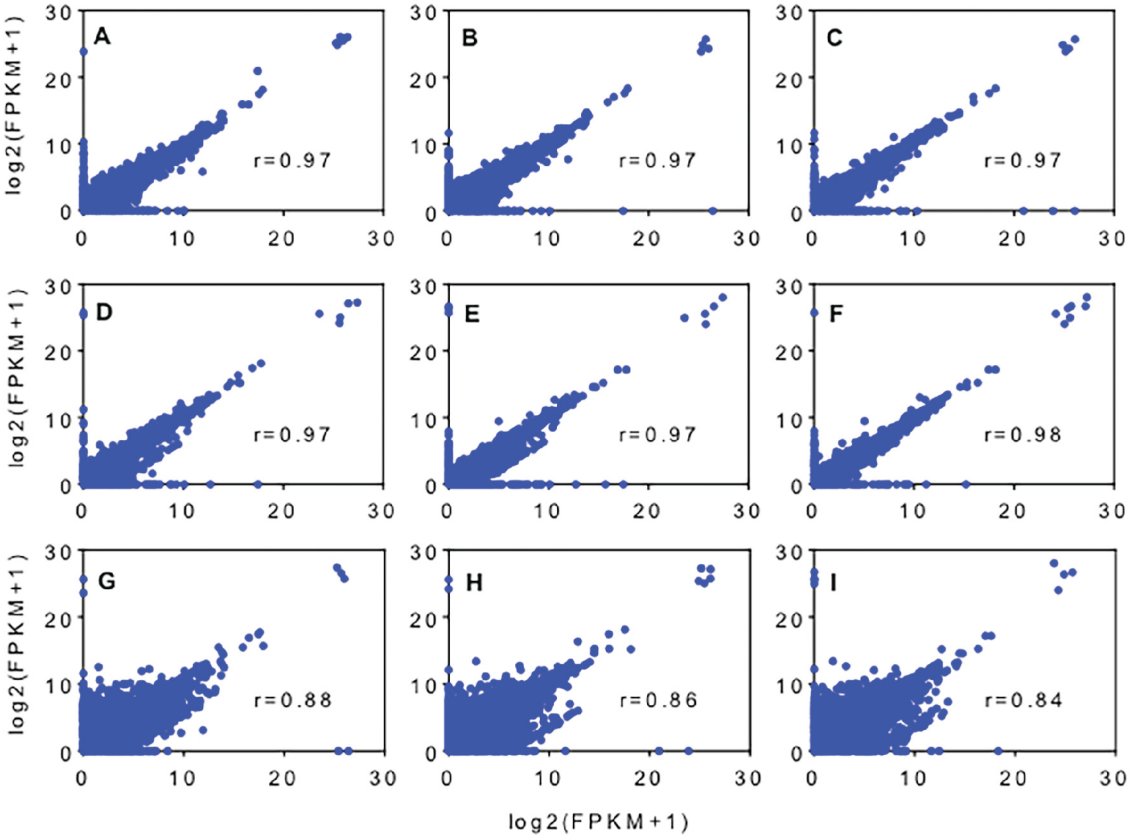

Supplementary Figure 2: Reproducibly of pHCC gene expression profiles. Each panel shows the Pearson's correlation of gene expression profiles between. **(A)** pPH1 and pPH2 cells; **(B)** pPH1 and pPH3 cells; **(C)** pPH2 and pPH3 cells; **(D)** pHCC1 and pHCC2 cell lines; **(E)** pHCC1 and pHCC3 cell lines; **(F)** pHCC2 and pHCC3 cell lines; **(G)** pPH1 cells and pHCC1 cell lines; **(H)** pPH2 cells and pHCC2 cell lines; **(I)** pPH3 cells and pHCC3 cell lines. Each blue dot represents an individual gene.

Supplementary Table 1: Elevated pHCC cell lines gene expression relative to pPH cells.

See Supplementary File 1

Supplementary Table 2: Reduced pHCC cell line gene expression relative to pPH cells.

See Supplementary File 1

Supplementary Table 3: Master regulators of genes with increased expression in Oncopig pHCC cell lines

Transcription factors	Number of upregulated target genes
KLF4	1,217
MZF1	927
AGGF1	767
ZNF503	751
ZCCHC14	734
NFE2L1	409

The number of target genes with elevated expression for each transcription factor is indicated for each cell line.

Supplementary Table 4: Master regulators of genes with increased expression across 18 human HCC cell lines.

See Supplementary File 1

Supplementary Table 5: Validation of RNA-seq results

Gene symbol	qRT-PCR (log2 FC)	RNA-seq (log2 FC)
ADAM8	2.326	4.991
FZD7	1.167	2.341
HIF1A	1.421	2.601
NFE2L3	1.697	2.977
NR4A2	1.416	2.726
SDCBP2	2.395	5.539
BHMT	-5.225	-5.177
A1BG	-5.373	-5.511
KLK3	-4.209	-3.794
MST1	-4.053	-4.174
ACKR1	-4.407	-4.194
F2	-4.823	-4.764
UBD	-5.264	-5.282
GATM	-3.958	-3.980
NKG7	-5.024	-5.015
EXTL1	-3.921	-4.045

Genes displaying increased (*ADAM8, FZD7, HIF1A, NFE2L3, NR4A2, SDCBP2)* and reduced expression (*BHMT, A1BG, KLK3, MST1, ACKR1, F2, UBD, GATM, NKG7, EXTL1*) in pHCC cell lines based on RNA-seq analysis. Relative transcript levels (qRT-PCR (log2 FC) fold change) under two different conditions was determined by qRT-PCR by measuring Ct values of each gene in the pHCC cell lines and the pPH cells and that of a housekeeping gene, *GAPDH*. The difference between the Ct value of a gene for pHCC cell lines and pPH cell lines was normalized to the Ct value of *GAPDH* (ΔΔCt) and expressed as 2^-ΔΔCt to give the log2 FC. RNA-seq (log2 FC) is the ratio of the transcript levels (fold change) expressed as FPKM of a gene under two different conditions as determined by RNA-seq analysis and converted to logarithm base 2.

Supplementary Table 6: Sequences of qPCR primers used.

See Supplementary File 1

Supplementary Table 7: RNA-seq sequencing depths for each cell line

Oncopig	Cells	Number of reads
1	pPH cells	28, 385, 664
	pHCC cell lines	31, 047, 086
2	pPH cells	30, 286, 514
	pHCC cell lines	32, 296, 259
3	pPH cells	23, 391, 879
	pHCC cell lines	29, 239, 571
